# Quinine in Tetanus

**Published:** 1881-07

**Authors:** 


Quinine in Tetanus.—We called attention professionally some years ago, in an article in the *Medical and Surgical Reporter* of Philadelphia, to the use of quinia in the treatment of tetanus during our civil war, and desire to say a few words now in regard to its great value in that formidable trouble, which might well be classed with the opprobia of our vocation. In a previous issue of this journal, we spoke of quinia, as the *anti-periodic*, *par excellence.* Having tried most of the materia medica with generally unsatisfactory results, and believing that the trouble was not entirely beyond the resources of our art, and knowing that quinia was possessed of undoubted antiperiodic proportions, and that its potent and oft repeated use had given satisfactory results in almost every form of periodical disease, I resolved upon its use in tetanus, for the purpose of widening or increasing the intervals between the spasms in that trouble, if no other good should result, until amputation could be satisfactorily performed, for the removal of the traumatism, upon which its symptoms depended. As the chief surgeon in a large hospital in Mobile, Alabama, and as active engagements were occurring in its vicinity quite often, the opportunity was not long delayed for putting my theoretical views into actual practice. I may add in passing, that amputation of the offending member or part, had already been practiced as a *dernier* resort in two cases, after the utter failure of all other means at my command to afford relief, before the idea of the use of quinine suggested itself to my mind. But those operations were unsuccessful. In applying the idea practically, the patient was brought sufficiently under the influence of opium or morphia, by the mouth or hypodermically, to lessen the irritability of the nerves distributed to the traumatism, and when necessary, the impression was carried to narcotism, more or less complete ; after which, quinia by the mouth or hypoderm, in large doses, so as to increase the space of time between the paroxysms was given. When the intervals had been sufficiently extended to allow of amputation, or the removal of the traumatism under anæsthesia and the dressing of the stump: a full dose of opium or morphia, in conjunction with a large dose of quinine was given, and repeated for three or four doses every two hours, as necessary ; with complete success in three amputations—all that were performed in this way, in traumatic tenanus! The late Dr. J. C. Nott, Dr. Henderson, of the British Army, and Dr. Armstrong assisted, and witnessed two of these amputations, and were highly gratified with the results. The first case was operated upon in the presence of my surgical assistants only; but the result proving entirely satisfactory. Other gentlemen were invited, both to see and examine the cases prior to my plan of treatment, and also to be present at the operations. Attention is directed to these cases as additional evidence of the great value of quinine, as an antiperiodic. More of its valuable properties will be brought forward from time to time, as occasion favors.

